# Time-course changes in left ventricular myocardial deformation in STZ-induced rabbits on velocity vector imaging

**DOI:** 10.1186/1476-7120-12-17

**Published:** 2014-05-29

**Authors:** Shi Zeng, Tao Jiang, Qi-chang Zhou, Lianghui Yuan, Jia-wei Zhou, Dan-min Cao

**Affiliations:** 1 Department of Ultrasonography, the Second Xiangya Hospital, Central South University, No.139 Middle Renmin Road, Changsha, Hunan 410011, P.R. China; 2 Department of Ultrasonography, the First Hospital of Hunan University of Chinese Medicine, Changsha, Hunan 410011, P.R. China

**Keywords:** DM, Myocardial deformation, VVI, STZ-induced rabbits

## Abstract

**Objectives:**

To clarify the time-course changes in left ventricular myocardial deformation using velocity vector imaging and to provide insights into our understanding of the cardiac pathophysiology in diabetes mellitus.

**Methods:**

Thirty New Zealand white rabbits were randomly divided into either the control group (n = 10) or the diabetes mellitus (DM) group (induced with STZ, n = 20). For the myocardial deformation studies, echocardiography and syngo-vector velocity imaging (VVI) were performed at baseline and after 2, 4, 8, and 12 weeks in all of the rabbits. The left ventricular (LV) global longitudinal and circumferential strain and strain rate were measured. For histomorphological study of the heart structure, 2 of the STZ-induced rabbits were killed at 2, 4, 8, and 12 weeks. Routine hematoxylin and eosin staining was performed.

**Results:**

At 2 weeks, the global longitudinal strain (GLS), systolic strain rate (GLSRs), and diastolic strain rate (GLSRd) were significantly lower in the DM group compared with the control group (-18.16% versus -24.00%, -1.86 s^-1^ versus -2.49 s^-1^, 1.93 s^-1^ versus 2.42 s^-1^, respectively, P < 0.05), while, compared with the control group, the global circumferential strain (GCS), systolic strain rate (GCSRs), and diastolic strain rate (GCSRd) in the DM group were significantly decreased (-12.77% versus -23.31%, -1.31 s^-1^ versus -2.20 s^-1^, 1.41 s^-1^ versus 2.15 s^-1^, respectively, P < 0.05) at 8 weeks. With the progression of untreated diabetes, the histoanatomical alterations intensified gradually beginning at 2 weeks.

**Conclusions:**

The progressive impairments in LV myocardial deformation and structure occurred early in diabetic rabbits with normal LV ejection fraction (EF), FS, and E/A. VVI could be used to evaluate subtle cardiac dysfunction in the early phase of DM.

## 

Diabetes mellitus is among the most common chronic diseases worldwide. It has been estimated that there will be 340 million people suffering from DM by 2030 [1]. Cardiovascular complications are the major morbidity and cause of mortality in type 1 diabetes mellitus (T1DM). Moreover, once cardiovascular disease is clinically manifested, DM remains an independent risk factor for adverse outcomes [2,3]. Early detection of diabetic heart disease is important for timely interventions that prevent the future development of heart failure. Therefore, it is crucial to comprehensively evaluate cardiac dysfunction during the early development of T1DM.

Vector velocity imaging (VVI) is a novel speckle tracking-based technique for analyzing myocardial tissue motion and velocity, without the limitations of Doppler echocardiography [4-7]. This imaging is achieved via a combination of speckle tracking, mitral annulus motion, tissue-blood border detection, and the periodicity of the cardiac cycle. It enables rapid and accurate quantitative assessment of global and regional myocardial function, in terms of myocardial velocities, strain (myocardial tissue deformation), and strain rate (deformation rate). The technique provides an intuitive analysis of myocardial mechanics. Few studies have used speckle derived strain echo methods as VVI and two-dimensional strain in type 1 diabetes [8-11], but no reports have shown a trend in myocardial deformation changes in the very early stages of T1DM. The studies by Wei Z et al. [8,9] showed that circumferential strain and strain rate decreased in diabetic rats at 12 weeks after STZ induction. The study by Nakai H et al [12] showed that diabetic duration was the only independent confounder in the reduction of longitudinal strain. Thus, we hypothesized that cardiac dysfunction perhaps already exists in the very early stages of T1DM.

The purposes of this study were to clarify the time course changes in left ventricular myocardial deformation in STZ-induced diabetic rabbits using VVI, to provide insights into the pathophysiology of diabetic cardiomyopathy and to demonstrate the validity of VVI in identifying heart structure alterations in T1DM.

## Materials and methods

### Experimental animals

The experimental design was approved by the Central South University Animal Care and Use Committee. The institution’s guidelines for the care and use of laboratory animals were observed.

STZ, a nitrosourea derivative that is able to increase DNA alkylation, is the most commonly used drug to induce type 1 diabetes in animal models [13]. GLUT2-mediated transport of STZ in β-cells leads to multiple DNA strand breaks [14] and subsequent activation of poly-ADP-ribosylation. These events are followed by depletion of intracellular NAD and ATP, and the insulin-secreting cells undergo necrosis [15]. This immune response resembles the pathophysiology of human type 1 diabetes. Therefore, STZ-diabetic animals, including rodents, rabbits, monkeys, and pigs, have been used for decades to investigate the systemic cardiovascular features of type 1 diabetes.

The animals used were 30 male New Zealand rabbits with a mean body mass of 2000 g. We chose only male rabbits for the study because in our preliminary study, compared with the female rabbit model, the male rabbit model tended to have a higher success rate and a lower mortality rate. The rabbits were randomly divided into the DM group (n = 20) and the control group (n = 10). Diabetes was induced by a single injection of STZ (65 mg/kg) (1% STZ solution, diluted with 0.1 M citrate buffer, pH 4.4, before injection) via the ear vein after a 12-hour fast. The control animals were injected with a similar volume of citrate buffer solution. All of the rabbits were fed a standard diet and were provided water ad libitum. Glucose concentrations and body mass were monitored weekly and at the time of kill (after 2, 4, 8, and 12 weeks of disease induction).

### Echocardiography

All of the rabbits underwent routine echocardiography, tissue Doppler imaging and syngo-VVI prior to and at 2, 4, 8, and 12 weeks after the injections. These procedures were performed by a single experienced operator (T.J.) who was blinded to the group status of the subjects, the stage of the study, and the previous measurements. All of the rabbits were anesthetized by intraperitoneal injection of 3% sodium pentobarbital (1 mL/kg) and were placed in a supine position with the anterior chest hair removed. A complete echocardiogram was performed using a Siemens Acuson S2000 system (Mountain View, CA, USA) coupled to a 10V4C transducer. Multiple two-dimensional (2D) views of the heart were obtained to evaluate the cardiac anatomy. At least three consecutive heartbeats were analyzed, with the values represented as the mean values. Heart rate, LA diameter, LV end-systolic and end-diastolic diameters, interventricular septum (IVS) thickness, and left ventricular posterior wall (LVPW) thickness were measured by M-mode echocardiography. Left ventricular ejection fraction (LVEF) was calculated by the modified Simpson’s method. Transmitral flow velocities were obtained by pulsed-wave Doppler echocardiography by positioning a sample volume at the level of the mitral tip. The peak velocities during early diastole (E) and late diastole (A) were measured, and their ratio (E/A ratio) was calculated.

### Tissue doppler imaging

TDI of the mitral annulus was obtained from the apical 4-chamber view. The sample volume was placed sequentially at the lateral and medial mitral annulus. The early diastolic annular velocitiy (e’) was measured and analyzed as the average of the medial and lateral annulus. The mitral inflow E velocity to tissue Doppler e’ (E/e’) ratio, was calculated to estimate LV filling pressures.

### VVI Image acquisition and offline analysis

**Figure 1:**
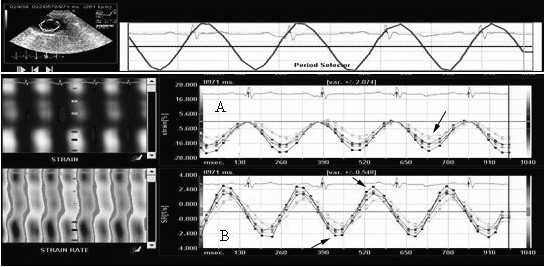
**Representative strain and strain rate curves derived from VVI in the short-axis view.** On the strain curve **(A)**, the peak systolic strain was measured (arrow). On the strain rate curve **(B)**, the peak systolic strain rate (negative value) and diastolic strain rate (positive value) were measured.

ECG leads were connected to the paws. Digital dynamic magnified loops of long- and short-axis views, incorporating at least three complete cardiac cycles, were recorded. The images were stored in DICOM format and were analyzed off-line using Syngo VVI software. The endocardium was manually traced, with the blood interface of the left ventricles at end systole. On the three long-axis (apical 2-, 3-, and 4-chamber) views, the endocardium was manually traced, beginning with the free wall edge of the atrioventricular valve annulus, extending to the apex, and returning to the septal edge of the atrioventricular valve annulus, while on the three short-axis (at the mitral valve, papillary muscle, and apex level) views, the endocardium was manually traced beginning with the anteroseptum and returning to the original location in a clockwise direction. After placing the index at the apex for the long-axis views and at the middle of the cycle for the short-axis views, VVI myocardial deformation analysis was then performed. Cardiac strain and strain rates for systole and diastole were automatically calculated and were displayed in a six-segment model. The peak systolic strain and the peak systolic and diastolic strain rates were measured (Figure 1). Each value was measured as the mean of three cardiac cycles.

Global longitudinal strain (GLS), systolic strain rate (GLSRs), and diastolic strain rate (GLSRd) were displayed as the average of all 18 segments of the three long-axis (apical 2-, 3-, and 4-chamber) views, while global circumferential strain (GCS), systolic strain rate (GCSRs), and diastolic strain rate (GCSRd) were displayed as the average of all 18 segments of the three short-axis (at the mitral valve, papillary muscle, and apex) views.

### Interobserver and intraobserver variability

Interobserver variability was assessed between two independent investigators (T.J. and L.Y.) on 20 randomly selected data sets. To assess intraobserver variability, 10 randomly selected subjects were blindly analyzed three times by one investigator (T.J.) within 1 week. Intra- and inter-observer variabilities were then calculated as the absolute differences between the corresponding two measurements as the percentages of their means.

### Histomorphological study of the heart

Two of the STZ-induced rabbits were killed at 2, 4, 8, and 12 weeks after echocardiography. Histomorphological examination was performed after fixing the hearts of the rabbits in 10% formalin and processing and embedding them in paraffin. Tissue blocks were sectioned at a thickness of 5 μm and were stained with Harris hematoxylin and eosin (H&E).

### Data analysis

The data are presented as the means ± standard deviations (SDs) or are plotted as the means with SD bars. The time-course changes in the DM group were assessed using Scheffe’s test for multiple comparisons after one-way analysis of variance. The unpaired two-tailed Student's t-test for continuous variables was performed between the DM and control groups at the same time points. The differences between the longitudinal and circumferential parameters in the control group were assessed by the paired t-test. A two-sided P < 0.05 was considered statistically significant. All of the statistical analyses were performed using the SPSS software package (SPSS for Windows; SPSS Inc., Chicago, IL, USA).

## Results

### General conditions

The rabbits in the DM group exhibited less activity (tended to blink and move about less frequently), loss of energy, and the presence of clinical symptoms of diabetes beginning on the fifth day post-injection. In the DM group, one rabbit died within 8 hours after injection, and another died at 2 weeks. The rabbits in the control group remained healthy, and none died. Thus, 10 rabbits in the control group and 18 rabbits in the DM group were included in the analysis.

No significant baseline weight differences were observed between the DM and control groups (2.03 ± 1.33 kg vs. 2.03 ± 1.14 kg, P > 0.05). As expected, the fasting blood glucose in the DM group increased gradually and remained greater than 300 mg/dL beginning one day after injection, while the body mass in the DM group was significantly lower than that in the control group at 12 weeks after injection (3800.39 ± 23.42 g vs. 3392.23 ± 11.51 g, P < 0.05).

### Echocardiogram

In the control group, the echocardiographic parameters, such as heart rate, LA diameter, LVESD, LVEDD, IVS thickness, PW thickness, FS, EF, and E/A, E/e’remained unchanged (Table 1).

**Table 1:** General conditions and routine echocardiographic parameters in the control and DM groups ()

	**Control group**					**DM group**				
	**Baseline**	**2 weeks**	**4 weeks**	**8 weeks**	**12 weeks**	**Baseline**	**2 weeks**	**4 weeks**	**8 weeks**	**12 weeks**
Glucose (mg/dL)	92.34 ± 10.98	91.44 ± 16.56	93.06 ± 11.16	91.80 ± 14.76	87.66 ± 12.78	91.08 ± 10.08	409.14 ± 74.34*	409.50 ± 81.72*	418.5 ± 88.56*	424.44 ± 101.7*
HR (bpm)	235 ± 11	215 ± 10	219 ± 13	230 ± 14	227 ± 8	219 ± 6	225 ± 18	241 ± 10	239 ± 13	212 ± 8
LA (mm)	7.82 ± 0.61	7.58 ± 0.79	8.03 ± 0.62	7.69 ± 0.82	7.76 ± 0.47	7.52 ± 0.74	7.49 ± 0.62	7.78 ± 0.54	7.62 ± 0.50	11.57 ± 0.84*
LVDd (mm)	11.83 ± 1.10	11.65 ± 1.07	11.72 ± 1.82	11.47 ± 1.63	11.84 ± 1.40	11.92 ± 2.26	11.48 ± 0.91	11.57 ± 1.50	11.68 ± 1.34	13.69 ± 0.92*
LVDs (mm)	8.53 ± 1.35	8.29 ± 1.22	8.62 ± 1.10	8.71 ± 0.86	8.44 ± 1.52	8.29 ± 1.85	8.31 ± 1.03	8.49 ± 1.30	8.22 ± 1.53	10.38 ± 1.26*
LVPW (mm)	2.35 ± 0.43	2.24 ± 0.43	2.37 ± 0.39	2.45 ± 0.28	2.41 ± 0.51	2.40 ± 0.61	2.52 ± 0.23	2.48 ± 0.51	2.45 ± 0.59	3.36 ± 0.45*
IVS (mm)	2.29 ± 0.32	2.32 ± 0.36	2.30 ± 0.41	2.32 ± 0.42	2.25 ± 0.50	2.21 ± 0.62	2.34 ± 0.53	2.30 ± 0.57	2.33 ± 0.48	3.29 ± 0.39*
EF (%)	63.56 ± 5.53	60.75 ± 6.22	65.39 ± 3.82	62.77 ± 8.20	65.43 ± 4.82	62.58 ± 5.77	63.34 ± 4.92	61.89 ± 6.58	63.87 ± 4.52	44.77 ± 5.42*
FS (%)	31.59 ± 5.87	35.72 ± 4.39	35.01 ± 4.62	33.69 ± 5.28	32.49 ± 6.31	30.64 ± 5.52	32.41 ± 2.56	31.78 ± 2.69	32.52 ± 2.53	24.83 ± 4.48*
E/A	1.56 ± 0.51	1.4 ± 0.27	1.67 ± 0.33	1.55 ± 0.42	1.62 ± 0.22	1.53 ± 0.61	1.60 ± 0.48	1.57 ± 0.60	1.60 ± 0.23	0.75 ± 0.38*
E/e’	3.1 ± 1.2	3.0 ± 0.6	3.0 ± 1.0	3.1 ± 0.8	3.0 ± 1.2	3.1 ± 1.1	3.3 ± 1.3	3.8 ± 1.8	6.1 ± 3.5*	8.7 ± 2.7*

*P < 0.05 versus the control group at the same time point.

In the DM group, before 12 weeks, the above-mentioned parameters also remained constant except E/e’ increased at 8 weeks, while after 12 weeks, compared with the control group, LA diameter, LVESD, LVEDD, IVS thickness, and PW thickness were increased; FS, EF, and E/A were decreased (all P < 0.05).

### Velocity vector imaging parameters

**Figure 2:**
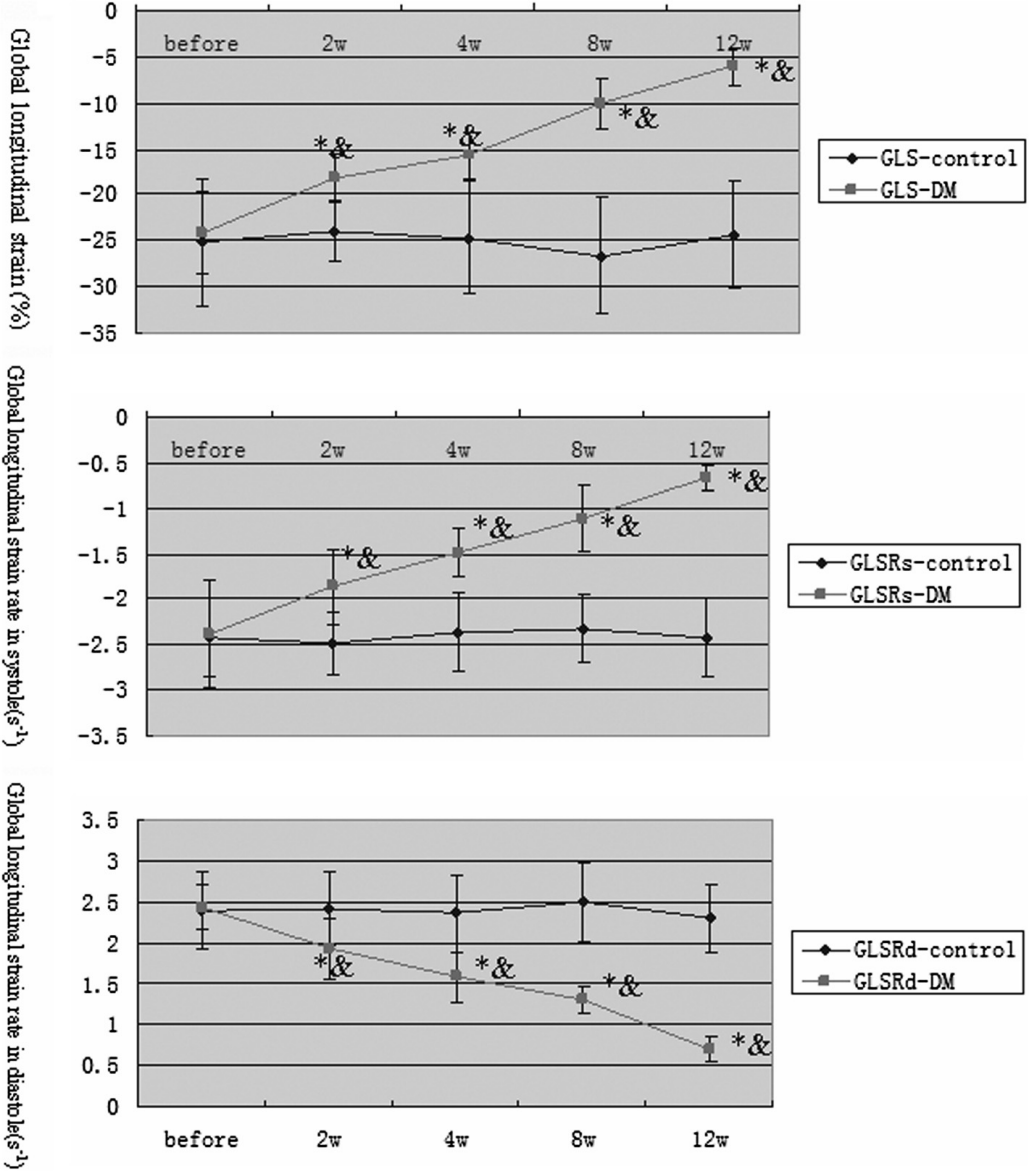
**Time course changes in global longitudinal strain and strain rate.** *P < 0.05 versus the control group; &P < 0.05 versus the DM group based on ANOVA.

**Figure 3:**
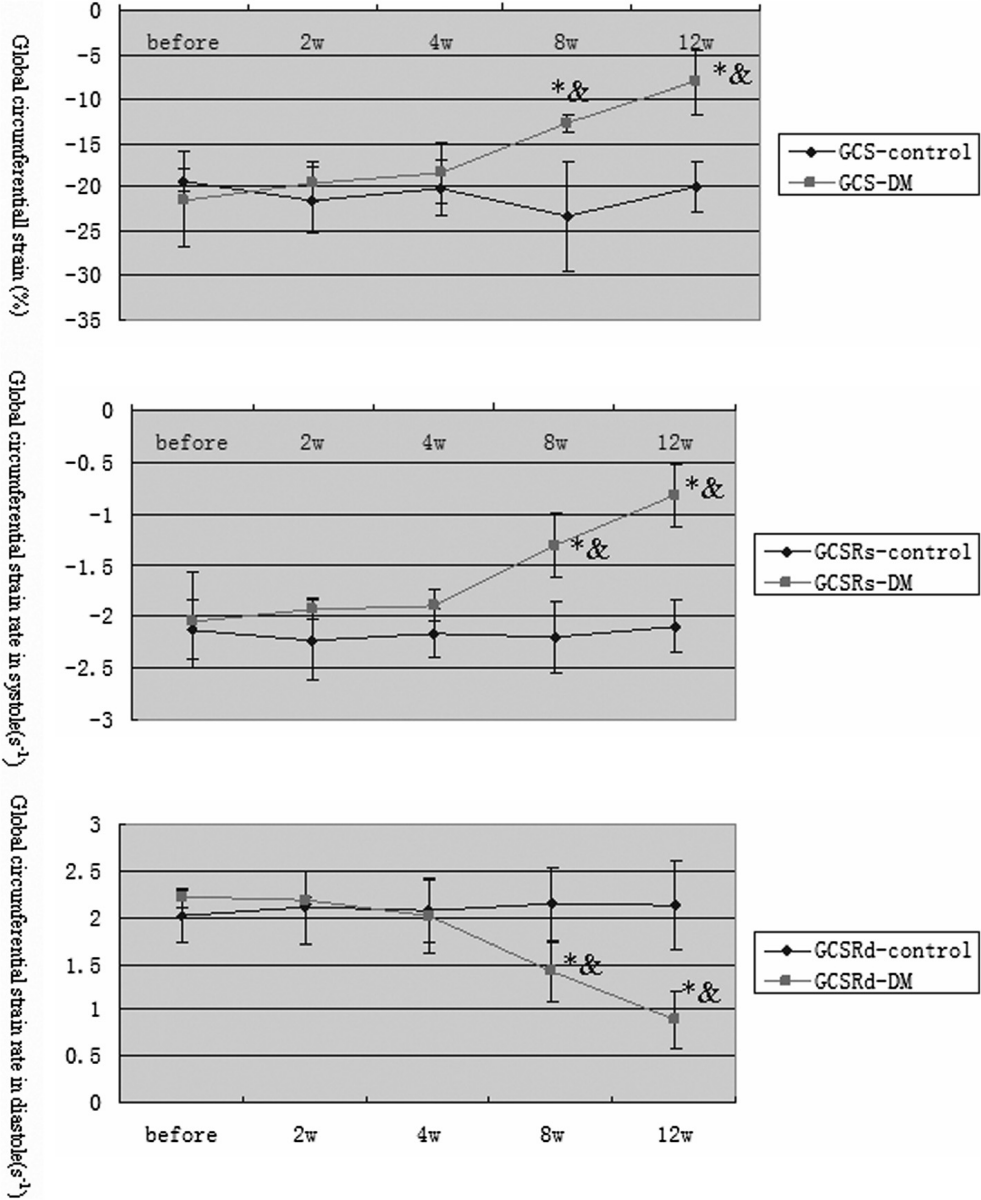
**Time course changes in global circumferential strain and strain rate.** *P < 0.05 versus the control group; &P < 0.05 versus the DM group based on ANOVA.

In STZ-induced rabbits, 2 weeks later, global longitudinal parameters, such as GLS, GLSRs, and GLSRd, were significantly lower than those pre-injection (P < 0.05) and those in the control group (P < 0.05) (Figure 2). At 8 weeks, compared with earlier time points in the DM and control groups, the global circumferential parameters, such as GCS, GCSRs, and GCSRd, in the DM group were significantly decreased (P < 0.05, Figure 3). Moreover, as time passed, all of the VVI parameters decreased gradually with significance (P < 0.05).

### Histomorphological study

**Figure 4:**
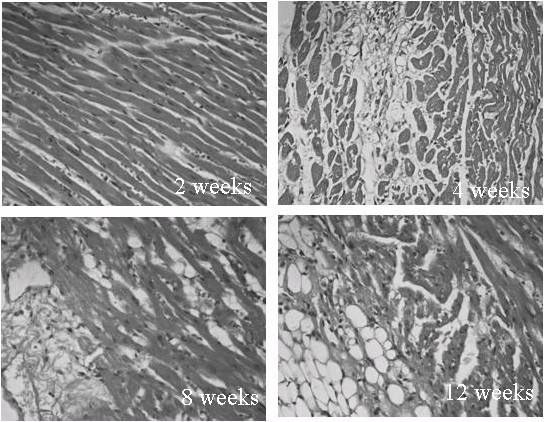
**Myocardial histoanatomical alterations in STZ-induced DM rabbits.** The heart sections of STZ-induced rabbits 2 weeks following diabetes induction showed mild edema and minor vacuolization in the cytoplasm. After 4 weeks, obvious edema, more vacuolization in the cytoplasm, and a few hemorrhages were observed. Eight weeks later, in addition to moderate vacuolization and hemorrhage, histiocyte proliferation and interstitial fibrosis appeared. After 12 weeks, degenerative changes in the nuclei, severe histiocyte proliferation, interstitial fibrosis, and collagen deposits were observed.

The heart sections of STZ-induced rabbits showed mild edema and vacuolization in the cytoplasm after 2 weeks. After 4 weeks, obvious edema, more vacuolization in the cytoplasm, and a few hemorrhages were observed. Eight weeks later, in addition to moderate vacuolization and hemorrhage, histiocyte proliferation and interstitial fibrosis appeared. After 12 weeks, degenerative changes in the nuclei, severe histiocyte proliferation, interstitial fibrosis, and collagen deposits were observed (Figure 4).

### Observer variability

The interobserver variability in the longitudinal and circumferential directions was 13% and 15% for strain, 6% and 7% for the systolic strain rate, and 8% and 10% for the diastolic strain rate, respectively. The intraobserver variability was 9% and 10% for strain, 5% and 6% for the systolic strain rate, and 6% and 5% for the diastolic strain rate in the longitudinal and circumferential directions, respectively.

## Discussion

The major findings of this study can be summarized as follows: 1) in STZ-induced diabetic rabbits, global strain and strain rates derived from VVI were significantly reduced a few weeks after the induction of diabetes and before the onset of detectable changes in routine echocardiographic parameters, such as EF, FS, and E/A; and 2) progressive reductions in longitudinal strain and strain rates started 6 weeks earlier than those in circumferential strain and strain rates.

In the present study, conventional echocardiographic parameters remained within normal ranges until 12 weeks after the induction of diabetes, but profound decreases in strain and strain rates appeared on VVI by 2–8 weeks after diabetes induction. These findings suggest that both systolic and diastolic LV myocardial performance were impaired in the early stage of T1DM, which was in agreement with our histoanatomical results.

Diastolic dysfunction (DD) is the most frequent echocardiographic finding in asymptomatic T1DM patients with normal LVEF. DD in T1DM have mainly been examined by standard echocardiography parameters like E/A, and also by tissue Doppler imaging like E/e’ [11-16]. Routine diastolic parameter E/A cannot distinguish pseudonormal and normal patterns of transmitral inflow because of its preload dependence. The ratio E/e’ may be extremely useful for characterizing DD and LV filling pressure because early diastolic peak velocity (e’) of the mitral annulus reflects the rate of myocardial relaxation and is relatively insensitive to preload effects [17] STZ-induced T1DM rats and mice also exhibited impairments in diastolic function, as shown on Doppler echocardiography (E/A, deceleration time, and IVRT) and cardiac catheterization (elevated LV end-diastolic pressure, LVEDP) in vivo [18-20]. Our study showed E/A decreased at 12 weeks, E/e’ increased and circumferential diastolic strain rate decreased at 8 weeks, longitudinal diastolic strain rate decreased at 2 weeks. The finding indicated diastolic strain rate might be more sensitive to impaired left ventricular diastolic function. Muranaka et al [21] demonstrated that early diastolic circumferential strain rates were reduced in diabetic patients using strain rate imaging. Wei Z et al [8,9] reported that 12 weeks after STZ induction, circumferential diastolic strain rates of the mid-level wall decreased mildly from 3.53^-1^ to 2.47^-1^. Hyperglycemia, insulin resistance, sympathetic overdrive, endothelial dysfunction, abnormalities of the angiotensin-renin system (ARS), and LV remodeling/hypertrophy may induce diastolic dysfunction (DD) and impairment of the coronary microcirculation and the microvascular alterations may worsen DD in DM [17].

Although diastolic dysfunction has been described as an early stage of diabetic heart disease progression in patients with normal LVEFs, preclinical systolic alteration has also recently been described by strain. Labombarda F’s study [10] demonstrated GLS was significantly lower in the T1DM children and correlated with HbA1c. Sveen KA [11] also reported LV longitudinal strain was significantly reduced in the patients with T1DM compared to controls. In an experimental rat study, Weytjens C et al [22] detected, at 6 weeks after STZ injection, a lower myocardial velocity and systolic strain rate and delayed time-to-peak deformation on Doppler myocardial imaging. Wei Z et al [8,9] demonstrated that 12 weeks after STZ induction, the circumferential strain of the mid-level wall on the LV short-axis in diabetic rats decreased mildly from -13.51% to -11.02%, and the systolic circumferential strain rates decreased mildly from -3.46^-1^ to -2.34^-1^. However, our study showed that global circumferential strain decreased profoundly, from -21.49% to -8.03%, and systolic global circumferential strain rates decreased from -2.04^-1^ to -0.82^-1^. Additionally, global longitudinal strain and systolic strain rates decreased substantially after 12 weeks, from -24.19% to -6.09%, and -2.39^-1^ to -0.66^-1^. Differences between the study animals and in the methods of measuring strain/strain rate may account for the discrepancies between our study and that by Wei Z et al. However, we were still unable to determine why DM induced a mild form of cardiomyopathy in other animal studies but induced severe dysfunction in our study.

Our study showed longitudinal strain, systolic strain rate and diastolic strain rate decreased in the early stage (even at 2 weeks) after STZ induction, suggesting impairment of both contractile and diastolic function in DM. Ernande L et al [23] reported that systolic alteration occurred in 28% of patients with DM with normal diastolic function and in 35% of patients with diastolic dysfunction. Systolic strain alteration can exist despite normal diastolic function, indicating that diastolic dysfunction should not be considered the first marker of the preclinical form of diabetic cardiomyopathy [23]. The studies on T2DM also showed both global and regional longitudinal strains were significantly reduced in patients with DM with respect to controls [24]. Galderisi M’s study [25] reported strain rate and strain were significantly lower in diabetics at low-dose and high-dose dobutamine, indicating altered contractile reserve in uncomplicated diabetes. Additionally, Stanton T et al. underwent a study on Prediction of all-cause mortality and demonstrated global longitudinal strain measurement was superior to EF and WMSI for the prediction of outcome and may become the optimal method for assessment of global LV systolic function [26].

The metabolic features [27] of STZ-induced DM animals include prompt development of profound hyperglycemia, modest hypertriglyceridemia, ketosis, and markedly reduced plasma insulin levels. Disturbances in metabolism of the heart have been implicated as important contributors to diabetic cardiac complications. Impaired excitation-contraction coupling (indicating by prolongation of action potential duration, slowed decay and reduced amplitude in Ca^2+^ transient, reduced Ca^2+^ sensitivity of contractile proteins, reduced Ca^2+^ store in sarcoplasmic reticulum), inefficient ATP production (indicating by increased free fatty acid uptake, suppressed glucose oxidation, mitochondrial dysfunction), reduced coronary flow reserve (indicating by microangiopathy, reduced microvascular density, impaired regulation of smooth muscle cell) and remodeling of extra-cellular matrix (indicating by increased collagen deposition, increased cross-link of collagen and laminin fibers) may cause left ventricular contractile dysfunction [28].

This study yielded another interesting finding, namely that GLS and GLSR in the DM group decreased six weeks earlier than the decrease in GCS and GCSR, suggesting that longitudinal deformation occurred early in the disease course, while circumferential deformation occurred relatively late in the disease course. This finding was in accordance with the document published by the American Society of Echocardiography, the European Society of Echocardiography, and the Japanese Society of Echocardiography [29]. Additionally, unlike longitudinal parameters, circumferential deformation serves as a marker of more advanced myocardial damage [30] and of prognosis [31].

## Conclusion

The present study demonstrated that DM rabbits with apparently normal LV dimensions, EFs, and E/A had impaired myocardial function as measured by the 2D speckle tracking-derived strain and strain rate. This finding indicates that velocity and strain rate are more sensitive and may be used for the detection of early LV dysfunction in diabetes. Moreover, impaired longitudinal myocardial deformation occurred earlier than circumferential dysfunction. As time passed, heart function and structure underwent progressive adverse changes.

### Limitations

First, because the myofibers are oriented parallel to the ultrasound beam in the septal and lateral regions on the short-axis views, the gray-scale quality can deteriorate in these segments, further compromising the accuracy of GCS and GCSR measurements. Second, the radial strain and strain rate could not be measured. The myocardial deformations in DM in the radial direction require further research.

## Competing interests

The authors declare that they have no conflict of interests.

## Authors’ contributions

SZ carried out the statistical analysis and drafted the manuscript. TJ performed the animal model and participated in the image collection. QZ conceived of the study, and participated in its design and coordination. LY participated in the statistical analysis. JZ carried out the histomorphological examination. DC helped to draft the manuscript. All authors read and approved the final manuscript.

## Authors’ information

Tao Jiang and Shi Zeng Both are the first authors.
